# Involuntariness of job changes is related to less satisfaction with occupational development in long-term breast cancer survivors

**DOI:** 10.1007/s11764-021-01035-5

**Published:** 2021-04-27

**Authors:** Kati Hiltrop, Paula Heidkamp, Clara Breidenbach, Christoph Kowalski, Anna Enders, Holger Pfaff, Lena Ansmann, Franziska Geiser, Nicole Ernstmann

**Affiliations:** 1 Center for Health Communication and Health Services Research (CHSR), Department for Psychosomatic Medicine and Psychotherapy, University Hospital Bonn, Bonn, Germany; 2 Center for Integrated Oncology (CIO Bonn), University Hospital Bonn, Bonn, Germany; 3 German Cancer Society (DKG), Berlin, Germany; 4 Federal Centre for Health Education (BZgA), Cologne, Germany; 5 Institute of Medical Sociology, Health Services Research, and Rehabilitation Science (IMVR), University of Cologne, Cologne, Germany; 6 Division for Organizational Health Services Research, Department of Health Services Research, School of Medicine and Health Sciences, Carl von Ossietzky University Oldenburg, Oldenburg, Germany; 7 Department for Psychosomatic Medicine and Psychotherapy, University Hospital Bonn, Bonn, Germany

**Keywords:** Breast cancer, Return to work, Occupational development, Involuntariness, Job changes, Social capital

## Abstract

**Purpose:**

Considering that breast cancer survivors (BCSs) have been dealing with unwanted job changes after diagnosis, this study aimed to investigate involuntary job changes (unwanted modifications in employment since diagnosis) and explore the association between job changes, involuntariness, and occupational development satisfaction in BCSs 5–6 years after diagnosis.

**Methods:**

Data were drawn from the mixed-methods breast cancer patients’ return to work (B-CARE) study. We surveyed 184 female BCSs who were working at the time of study enrollment during hospitalization (T1), 10 weeks after discharge (T2), 40 weeks after discharge (T3), and 5–6 years after diagnosis (T4) and used descriptive measures and stepwise linear regression models for data analysis.

**Results:**

The mean age of BCSs was 57 years. A total of 105 participants reported 410 job changes, of which 16.1% were reportedly (rather) involuntary. The most commonly reported involuntary changes were increased workload (15.2%) and increased scope of work (15.2%). In the final model, significant predictors of satisfaction with occupational development 5–6 years after diagnosis were age, state of health ΔT2–T3, state of health ΔT3–T4, and involuntariness of job changes.

**Conclusions:**

Although the number of job changes alone is not substantially associated with BCSs’ satisfaction with occupational development, experiencing involuntary job changes is. Sociodemographic, disease-related, and work(place)-related factors may influence occupational satisfaction among BCSs.

**Implications for Cancer Survivors:**

The findings indicate the importance of strengthening one’s ability to work as desired to prevent involuntary job changes and enable desired work participation in long-term support. The significance of workplace characteristics highlights the need for employers to encourage satisfying work participation.

**Trial registration number:**

German Clinical Trials Register (DRKS00016982), 12 April 2019

**Supplementary Information:**

The online version contains supplementary material available at 10.1007/s11764-021-01035-5.

## Introduction

In Germany, approximately 492,000 new cancer cases were diagnosed in 2016 [1]. The most common cancer type among females is breast cancer, with almost 70,000 newly diagnosed cases per year [1]. Screening programs and therapy improvements contribute to a 5-year survival rate of 88% among female patients with breast cancer [2]. Furthermore, a significant proportion of affected women (30%) are younger than 55 years old when diagnosed [3].

Work-related outcomes are especially important for working-age breast cancer survivors (BCSs), considering that work can give meaning, provide financial security, allow social participation [4], and positively influence their quality of life [5]. In recent years, work-related outcomes, such as the timing and determinants of return to work (RTW), of cancer survivors have been extensively researched [6–10]. Disease-, treatment-, and work-related aspects as well as sociodemographic and psychosocial aspects influence RTW [6–10]. Objective long-term work-related outcomes such as work performance, absenteeism, and job changes in cancer survivors have also been studied [11–13]. Bijker et al. [11] found that an improved general functional status is associated with less absence, higher productivity, and slightly higher chances of RTW among cancer survivors. According to a systematic review, cancer survivors within 5 years after diagnosis have higher absenteeism than nonaffected individuals [12]. Regarding job changes, more than half of cancer survivor participants reported at least one change 2 years after diagnosis [13]. A multicountry study by Torp et al. [14] described that 6–37% of employed cancer survivors underwent occupational changes up to 6 years following diagnosis, and given that changes in working time were analyzed separately, approximately one-quarter of these respondents reduced their working hours after diagnosis. Moreover, cancer survivors partially attributed changes such as reduced working hours, changed tasks, and changed employers to cancer disease experience [13, 14]. Older age, presence of comorbidities, treatment with chemotherapy, and disease progression were reportedly predictors of experiencing job changes [15]. Reduced physical and mental work abilities were associated with work changes [16].

The insight on how BCSs perceive and evaluate objective work-related outcomes, such as job changes, remains largely unknown. Although using more subjective measures is necessary to determine BCSs’ perspectives, only few studies exist. Mehnert and Koch [17] reported that work satisfaction is associated with sociodemographic characteristics such as older age, higher income, and health-related quality of life. Furthermore, lower levels of satisfaction with the vocational situation could predict no RTW among BCSs [18]. More research on BCSs’ evaluation of work-related outcomes is needed to (1) understand if experienced work-related outcomes are evaluated as burdensome and disadvantageous and to (2) determine the need for support from or improvements in the healthcare and social systems.

**Fig. 1:**
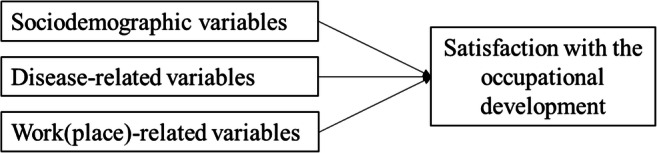
Research model

One aspect that might explain how disadvantageous work-related outcomes are for cancer survivors is probably their involuntariness. In the context of life-event research, stressful work-related events, particularly unintended job disruptions, directly and indirectly (mediated by coping and supportive resources) decrease mental health among working-age adult participants [19]. Currently, the association of involuntary job changes with work-related outcomes among cancer survivors has remained insufficiently researched. Initial studies reported the existence of unwanted job changes, such as demotion and changes in tasks and earnings, in BCSs after diagnosis [20]. However, the extent of experiencing involuntary work-related outcomes and the association of involuntariness with subjective work-related outcomes in cancer survivors are still unknown. Hence, this study aimed to (1) describe involuntary job changes and (2) explore the association between job changes, involuntariness, and satisfaction with the occupational development 5–6 years following a breast cancer diagnosis after controlling for sociodemographic, disease-related, and work(place)-related variables (Fig. 1).

## Methods

### Study design, sample, and data collection

**Fig. 2:**
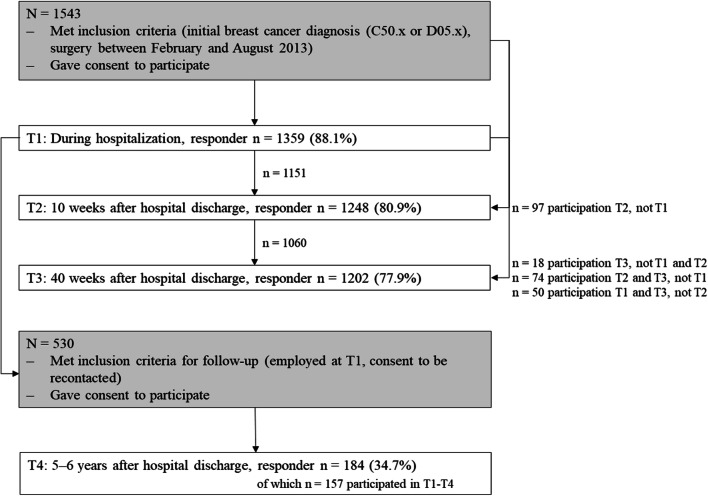
Flow of participants. Note: Respondents consisted of breast cancer survivors who consecutively participated in every survey wave and those who participated at least once. Dropouts occurred because of nonresponse, death, or unverifiable addresses

In this “breast cancer patients’ return to work” (B-CARE) study with mixed methods, we used BCSs’ longitudinal data that were collected at four measurement time points: during hospitalization, 10 weeks after discharge, 40 weeks after discharge, and 5–6 years after discharge (T1: *n* = 1359; T2: *n* = 1248; T3: *n* = 1202; T4: *n* = 184, respectively). Figure 2 illustrates the flow of participants. Data from the first three measurement time points were acquired from the PIAT study (“Strengthening patient competence: Breast cancer patients’ information and training needs”). The PIAT study was conducted in Germany from 2013 to 2014 and recruited a representative sample of breast cancer patients from 60 randomly selected certified breast cancer centers. These breast cancer centers invited all patients who had their initial breast cancer diagnosis (C50.x or D05.x) and surgery between February 2013 and August 2013. After written consent was obtained, participants answered the first paper-and-pencil survey during hospitalization (T1). The same patients received two more surveys via post in the follow-up treatment phase approximately 10 weeks after hospital discharge (T2) and in the post-treatment phase 40 weeks after hospital discharge (T3). For the mixed-methods B-CARE project, 530 PIAT participants who were employed during their breast cancer diagnosis and who gave consent to be recontacted in case of a follow-up were invited by post to complete another survey (response rate, 35%). Medical, psychosocial, and sociodemographic characteristics (e.g., UICC TNM stage, number of comorbidities, level of fear of progression, and age) did not significantly differ between responders and nonresponders at T4 (analyses not shown). Some of the participants underwent semistructured interviews. All postal mailings were conducted following the total design method to enhance the response rate [21]. Detailed information on the study design and sampling process can be found elsewhere [22, 23]. The Ethics Committees of the Medical Faculty of the University of Cologne approved the PIAT study and the Ethics Committee of the Medical Faculty of the University of Bonn approved the B-CARE study.

### Measurements

#### Satisfaction with occupational development

Satisfaction with occupational development at T4 was the dependent variable. It was measured with the item “Overall, how satisfied are you with your occupational development since your first breast cancer diagnosis?” on a 5-point Likert scale; the higher the values, the higher the level of satisfaction (1, dissatisfied; 2, rather dissatisfied; 3, partly; 4, rather satisfied; 5, satisfied).

#### Involuntariness of job changes

The respondents reported job changes that occurred since their diagnosis. These job changes were the following: increased/decreased working time, increased/decreased scope of work, increased/decreased workload, increased/decreased payment, change of employer, change within employer, and retirement entry (caused by age, early retirement, or reduced earning capacity). Considering that job changes can occur several times within 5–6 years, the questionnaire allowed us to chronologically indicate which of these job changes had occurred up to six occasions (Table S1 shows an excerpt from the questionnaire). For every occasion, the participants were asked the same questions. They were asked to report changes that occurred at that point in time (multiple choices from the aforementioned changes), rate the involuntariness of the chosen changes, and specify the point in time (month, year). The total score of reported changes was calculated using the dichotomous variables for all changes at all time points, possibly ranging from 0 to 66. The respondents were also asked to evaluate the voluntariness of the job changes on every occasion on a 5-point Likert scale (1, voluntary; 2, rather voluntary; 3, partly; 4, rather involuntary; 5, involuntary); the higher the values, the higher the level of involuntariness. Then, we calculated the average of the maximum six involuntariness ratings. The sum of job changes and their averaged involuntariness were measured at T4 and used as independent variables.

#### Sociodemographic, disease-related, and work(place)-related variables

Sociodemographic variables such as age at T4 (continuous), marital status at T4 (“single/divorced/widowed,” reference: “married”), number of children at T4 (continuous), and vocational training at T1 (“lower vocational training,” reference: “higher vocational training”) served as independent variables. Lower vocational training included participants who did (not or not yet) complete vocational training, whereas higher vocational training included participants who completed university (of applied sciences) or master craftsman training.

Disease-related variables were recurrence since diagnosis at T4 (“yes”, reference: “no”) and a subjective evaluation of the state of health (1, bad; 2, less good; 3, good; 4, very good; 5, excellent) measured at T2, T3, and T4 according to an item of the SF-36 Health Survey questionnaire [24]. Changes in self-reported state of health from ΔT2–T3 and ΔT3–T4 were calculated. Furthermore, the Union for International Cancer Control (UICC) TNM staging [25] added by clinical personnel at T1 was included.

The work(place)-related variable social capital (T4) was defined in this study as “features of social organizations such as networks, norms, and social trust that facilitate coordination and cooperation for mutual benefit” [26] and can be measured in workplaces. The social capital of the workplace where the participants returned to was measured using the SOCAPO-E instrument [27]. The instrument has six items that measure different social capital elements: warm circle, mutual understanding, trust, common values, “we”-feeling, and mutual help and reciprocity (“In my workplace, the willingness to help one another is great.”). The items were scored on a 4-point Likert scale (1, strongly disagree; 2, somewhat disagree; 3, somewhat agree; 4, strongly agree) and then averaged.

All measures were pretested in interviews or focus groups, as described elsewhere [22].

### Analysis

The quantitative survey data were digitalized using the data-capturing software Teleform version 16 and checked for plausibility. The pseudonymized PIAT (T1–T3) and B-CARE (T4) data sets were merged into one data set according to the study ID of each participant.

Missing values of the metric variables were imputed with the expectation maximization (EM) algorithm prior to the main analyses, namely, health status (T2, T3, T4), age (T4), involuntariness of work changes ratings (T4), and satisfaction with occupational development (T4). If a variable or instrument has more than 30% of missing values, imputation was not applied [28]. The EM algorithm estimates missing data according to an iterative maximum-likelihood process and is recommended for preventing biases caused by not completely at random missing data processes [29, 30].

Missing data in the categorical and ordinal variables used for calculating the UICC TNM stage (T1), recurrence since diagnosis (T4), and vocational training (T1) were replaced with modal values [31]. Meanwhile, the remaining missing data were deleted listwise.

Initially, we analyzed the frequencies of job changes and their involuntariness descriptively. Next, the associations between job changes, their involuntariness, and satisfaction with the occupational development of BCSs 5–6 years after diagnosis were investigated using three linear regression models with stepwise addition of variables. The first model M1a consisted of the sociodemographic, disease-related, and work(place)-related variables; M2a integrated the number of job changes; lastly, M3a added the average involuntariness of job changes, thereby estimated according to those participants who experienced at least one job change. We additionally calculated the models M1b-M3b with nonimputed data (Table 3). The assumptions of no multicollinearity, no autocorrelation of residuals, and no perfect linearity were tested for and subsequently met.

All statistical data were analyzed using the IBM SPSS Statistics version 24.

## Results

### Descriptive results

The study enrolled 184 BCSs, with a mean age of 57 years. On average, the respondents had 1.5 children, and almost 73.0% of them were married. In general, different levels of vocational training were observed. Nearly two-thirds had lower vocational training. The mean UICC TNM stage was 1.4, and the majority (80.4%) did not have a relapse within 5–6 years after diagnosis. Table 1 shows the descriptive statistics of the sample.

**Table 1:** Descriptive statistics of the sample

		Imputed data				Nonimputed data			
		*n* (%)	Mean	Standard deviation	Min–max	*n* (%)	Mean	Standard deviation	Min–max
Sociodemographic variables									
Age in years (T4)			56.90	6.54	36–79		56.93	6.82	36–79
	Missing	0 (0)				15 (8.2)			
Marital status (T4)	Married	134 (72.8)				134 (72.8)			
	Unmarried	50 (27.2)				50 (27.2)			
	Missing	0 (0.0)				0 (0.0)			
Number of children (T4)			1.52	0.98	0–4		1.52	0.98	0–4
	Missing	0 (0.0)				0 (0.0)			
Vocational training (T1)	Lower training	117 (63.6)				106 (57.6)			
	Higher training	67 (36.4)				67 (36.4)			
	Missing	0 (0)				11 (6.0)			
Disease-related variables									
UICC TNM stage (T1)			1.43	0.73	0–4		1.48	0.78	0–4
	Missing	1 (0.5)				34 (18.5)			
Recurrence (up to T4)	No	148 (80.4)				145 (78.8)			
	Yes	36 (19.6)				36 (19.6)			
	Missing	0 (0)				3 (1.6)			
State of health (ΔT2–T3)			0.26	0.64	−2 to 2		0.27	0.68	−2 to 2
	Missing	0 (0)				26 (14.1)			
State of health (ΔT3–T4)			0.05	0.74	−2 to 2		0.06	0.76	−2 to 2
	Missing	0 (0)				20 (10.9)			
Work (place)-related variables									
Social capital (T4)			3.00	0.68	1–4		3.00	0.68	1–4
	Missing	22 (12.0)				22 (12.0)			
Number of job changes (up to T4)			2.23	3.20	0–22		2.23	3.20	0–22
	Missing	0 (0.0)				0 (0.0)			
Involuntariness of job changes, averaged (T4)			2.00	1.17	1–5		1.98	1.19	1–5
	Missing	79 (42.9)				84 (45.7)			

In total, 105 BCSs reported 410 job changes during the 5–6-year period after diagnosis. More than half of the respondents (57.0%) experienced at least one job change. The most common changes were decreased working time (19.8%), decreased payment (10.5%), and decreased workload (10.0%). Furthermore, 16.1% of the job changes were experienced rather involuntarily or involuntarily, affecting 9.8% of the participants. Among the involuntary job changes, increased workload (15.2%) and increased scope of work (15.2%) were the most often reported changes, followed by retirement entry caused by reduced earning capacity or early retirement (12.1%) and decreased working time (12.1%). Table 2 lists the descriptive results.

**Table 2:** Job changes in breast cancer survivors (BCSs) since diagnosis

	All job changes (*n* = 105 participants)		Involuntary job changes* (*n* = 18 participants)	
	*n*	%	*n*	%
Decreased working time	81	19.76	8	12.12
Decreased payment	43	10.49	5	7.58
Decreased workload	41	10.00	6	9.09
Increased workload	40	9.76	10	15.15
Retirement entry	40	9.76	8	12.12
Decreased scope of work	37	9.02	4	6.06
Increased scope of work	32	7.80	10	15.15
Increased payment	32	7.80	5	7.58
Increased working time	24	5.85	2	3.03
Change of employer	21	5.12	5	7.58
Change within employer	19	4.63	3	4.55
Total	410	100.00	66	100.00

Note: *Job changes rated as “rather involuntary or involuntary” on a 5-point Likert scale

### Multivariate results

Table 3 shows results of the three stepwise linear regression models. The model M1a, which included sociodemographic, disease-related, and work(place)-related variables, reached significance (*F* [9, 81] = 3.372, *p* < 0.01) and explained 18.9% of variance in satisfaction with the occupational development 5–6 years after diagnosis (adjusted *R*^2^). The variables age (*β* = 0.038, *t* = 2.365, *p* < 0.05), state of health ΔT2–T3 (*β* = 0.422, *t* = 2.998, *p* < 0.01), state of health ΔT3–T4 (*β* = 0.349, *t* = 2.420, *p* < 0.05), and social capital of the workplace where the respondents returned to (*β* = 0.270, *t* = 2.071, *p* < 0.05) had a significant positive association with occupational development satisfaction 5–6 years after diagnosis in BCSs. In contrast, marital status, number of children, vocational training, UICC TNM stage, and recurrence were insignificant in M1a.

**Table 3:** Results of the stepwise linear regression models

	**M1a imputed**				**M2a imputed**				**M3a imputed**			
	**Regression coefficient**	**Standard error**	***T*-value**	**Significance**	**Regression coefficient**	**Standard error**	***T*-value**	**Significance**	**Regression coefficient**	**Standard error**	***T*-value**	**Significance**
(Intercept)	1.072	0.979	1.094	0.277	0.834	1.015	0.821	0.414	2.076	0.964	2.153	0.034*
Sociodemographic variables												
Age in years (T4)	0.038	0.016	2.365	0.020*	0.038	0.016	2.408	0.018*	0.032	0.015	2.229	0.029*
Unmarried (T4) (reference: married)	0.135	0.224	0.600	0.550	0.103	0.227	0.455	0.651	0.207	0.207	1.001	0.320
Number of children (T4)	−0.177	0.102	−1.746	0.085	−0.176	0.102	−1.733	0.087	−0.176	0.092	−1.907	0.060
Lower vocational training (T1) (reference: higher vocational training)	0.283	0.194	1.464	0.147	0.302	0.195	1.549	0.125	0.340	0.177	1.925	0.058
Disease-related variables												
UICC TNM stage	0.002	0.128	0.015	0.988	−0.003	0.128	−0.020	0.984	−0.028	0.116	−0.243	0.808
Recurrence (up to T4) (reference: no recurrence)	−0.195	0.249	−0.786	0.434	−0.213	0.250	−0.855	0.395	−0.221	0.226	−0.978	0.331
State of health (ΔT2–T3)	0.422	0.141	2.998	0.004**	0.436	0.142	3.072	0.003**	0.440	0.129	3.428	0.001**
State of health (ΔT3–T4)	0.349	0.144	2.420	0.018*	0.353	0.144	2.446	0.017*	0.436	0.132	3.301	0.001**
Work(place)-related variables												
Social capital (T4)	0.270	0.130	2.071	0.042*	0.297	0.134	2.219	0.029*	0.198	0.124	1.602	0.113
Number of job changes (up to T4)					0.029	0.032	0.901	0.370	0.035	0.029	1.202	0.233
Involuntariness of job changes, averaged (T4)									−0.323	0.075	−4.299	0.000***
Adjusted *R*^2^	0.189				0.187				0.333			
	**M1b nonimputed**				**M2b nonimputed**				**M3b nonimputed**			
	**Regression coefficient**	**Standard error**	***T-*value**	**Significance**	**Regression coefficient**	**Standard error**	***T*-value**	**Significance**	**Regression coefficient**	**Standard error**	***T*-value**	**Significance**
(Intercept)	0.856	1.069	0.801	0.427	0.983	1.091	0.901	0.372	2.005	1.048	1.914	0.061
Sociodemographic variables												
Age in years (T4)	0.036	0.018	2.014	0.049*	0.036	0.018	1.992	0.052	0.032	0.016	1.951	0.057
Unmarried (T4) (reference: married)	0.396	0.240	1.652	0.105	0.450	0.254	1.770	0.083	0.558	0.235	2.372	0.022*
Number of children (T4)	−0.288	0.113	−2.559	0.014*	−0.291	0.113	−2.568	0.013*	−0.267	0.104	−2.564	0.013*
Lower vocational training (T1) (reference: higher vocational training)	0.640	0.214	2.996	0.004**	0.638	0.215	2.971	0.005**	0.638	0.197	3.246	0.002**
Disease-related variables												
UICC TNM stage	−0.064	0.129	−0.496	0.622	−0.069	0.130	−0.534	0.596	−0.102	0.119	−0.856	0.396
Recurrence (up to T4) (reference: no recurrence)	−0.820	0.283	−2.897	0.006**	−0.826	0.285	−2.899	0.006**	−0.835	0.261	−3.200	0.002**
State of health (ΔT2–T3)	0.485	0.149	3.252	0.002**	0.480	0.150	3.195	0.002**	0.515	0.138	3.735	0.000***
State of health (ΔT3–T4)	0.364	0.161	2.254	0.029*	0.353	0.163	2.169	0.035*	0.478	0.154	3.104	0.003**
Work(place)-related variables												
Social capital (T4)	0.387	0.131	2.956	0.005**	0.382	0.132	2.896	0.006**	0.267	0.126	2.123	0.039*
Number of job changes (up to T4)					−0.024	0.036	−0.667	0.508	−0.027	0.033	−0.834	0.408
Involuntariness of job changes, averaged (T4)									−0.264	0.081	−3.258	0.002*
Adjusted R^2^	0.423				0.417				0.511			

Note: * *p<*0.05; ** *p*<0.01; ***p<0.001

M2a, which additionally included the number of job changes, reached significance (*F* [10, 80] = 3.069, *p* < 0.01), with an adjusted *R*^2^ of 18.7%. The variables age (*β* = 0.038, *t* = 2.408, *p* < 0.05), state of health ΔT2–T3 (*β* = 0.436, *t* = 3.072, *p* < 0.01), state of health ΔT3–T4 (*β* = 0.353, *t* = 2.446, *p* < 0.05), and social capital of the workplace where the respondents returned to (*β* = 0.297, *t* = 2.219, *p* < 0.05) had a significant positive association with occupational development satisfaction of BCSs 5–6 years after diagnosis. In this model, marital status, number of children, vocational training, UICC TNM stage, recurrence, and number of job changes were insignificant.

M3a also integrated the averaged involuntariness of job changes and was estimated for participants with at least one job change since diagnosis. It also reached significance (*F* [11, 79] = 5.079, *p* < 0.001), with an adjusted R^2^ of 33.3%. The variables age (*β* = 0.032, *t* = 2.229, *p* < 0.05), state of health ΔT2–T3 (*β* = 0.440, *t* = 3.428, *p* < 0.01), state of health ΔT3–T4 (*β* = 0.436, *t* = 3.301, *p* < 0.01) had a significant positive association with occupational development satisfaction of BCSs 5–6 years after diagnosis. Conversely, higher levels of involuntariness (*β* = −0.323, *t* = −4.299, *p* < 0.001) had a significant negative association with occupational development satisfaction 5–6 years after diagnosis. Moreover, marital status, number of children, vocational training, UICC TNM stage, recurrence, social capital of the workplace where the respondents returned to, and number of job changes were insignificant in this model.

In comparing the results between the imputed data (M1a–M3a) and the nonimputed data (M1b–M3b) (Table 3), the regression coefficients were similar, except for the variables marital status, number of children, vocational training, and recurrence, which were smaller in the models with imputed data.

For reliability analysis, Cronbach’s alpha was calculated for the validated instrument social capital (Cronbach’s alpha, 0.94).

## Discussion

This study aimed to (1) describe BCSs’ involuntary job changes and (2) explore the associations between such job changes, involuntariness, and occupational development satisfaction 5–6 years after breast cancer diagnosis, while controlling for sociodemographic, disease-related, and work(place)-related variables.

In the descriptive results, more than half of the participants reported job changes 5–6 years after the diagnosis. This proportion is lower than that reported by Steiner et al. [13] in which 67% of cancer survivors in the USA experienced job changes within 2 years after diagnosis. Such variation may be explained by the fact that Germany has different employment laws and special protection for employees with disabilities (e.g., in terms of dismissal). In the multivariate results, the number of job changes alone does not significantly influence the BCSs’ satisfaction with their occupational development or enhance the exploratory power of the model.

According to the descriptive results, 16% of all job changes after breast cancer were involuntary or rather involuntary. The most commonly reported involuntary changes were increased scope of work, increased workload, and retirement entry. These changes suggest that meeting the (increasing) demands at work might be challenging for BCSs, forcing them to reduce their working time or retire (early retire or retire because of reduced earning capacity). Regarding the multivariate findings, involuntariness of job changes was negatively associated with BCSs’ satisfaction with their occupational development 5–6 years after diagnosis. After the inclusion of involuntariness in the model, the adjusted *R*^2^ increased by approximately 15%. Therefore, involuntariness can be an important barrier for the BCSs’ ability to work as desired and may be linked to other disadvantages, such as financial strain. Offering access to rehabilitation services for BCSs several years after their diagnosis might be crucial to help them meet the work demands and prevent involuntariness in the long run. These results can be discussed against the background of life-event research. Stressful work-related events, particularly involuntary job disruptions, decrease one’s well-being both directly and indirectly (mediated by coping and supportive resources) [19]. The present results underline that the quality of stress-inducing events is a more important indicator than the frequency [19]. Involuntariness in the work context might not only affect mental health but also the satisfaction with occupational development.

In linear regression model analysis, the sociodemographic, disease-related, and work(place)-related factors were associated with BCSs’ satisfaction with their occupational development 5–6 years after being diagnosed.

The present study showed that higher age is positively associated with BCSs’ satisfaction with their occupational development [17]. Meanwhile, marital status had no significant association. Literature on the relationship between marital status and work satisfaction seems to be varied. While Clark [32] found that married people are more satisfied with work, Gazioglu and Tansel [33] reported that work satisfaction is higher among unmarried individuals. Furthermore, Mehnert and Koch [17] described that BCS’ work satisfaction is associated with higher education, but the present study revealed that vocational training is not associated with higher levels of satisfaction with the occupational development. However, other studies also found that lower vocational training is associated with higher levels of work satisfaction [33]. Hence, the evidence seems ambiguous. Other indicators such as physical or mental strain of the job could be more suitable predictors of work satisfaction and satisfaction with the occupational development in BCSs, given that many suffer from long-term consequences of the disease and its treatment.

In a previous study, disease-related variables (cancer entity, stage, disease phase/remission, time since diagnosis, and treatments) were not associated with work satisfaction [17]. Consistent with these results, the UICC TNM stage and recurrence since the time of diagnosis did not show significant effects in the present analyses. In finding significant effects for subjective health status, subjective measures may be more suitable predictors for satisfaction with the occupational development than the objective measures. In the present study, an increase in the self-reported state of health from T2–T3 and T3–T4 was significantly associated with higher levels of satisfaction with the occupational development. These outcomes are in line with those of a former study that suggested an association between work satisfaction and health-related quality of life [17].

Regarding work(place)-related variables, a positive association was found between higher levels of social capital in the workplace where the participants returned to and satisfaction with the occupational development 5–6 years after diagnosis in M1a and M2a. This finding is consistent with the results of Ommen et al. [34], who found a positive association between social capital and work satisfaction among hospital-based physicians. Given that the association between social capital of the workplace and satisfaction with the occupational development is rarely studied, the discussion was extended to include findings on social support of the workplace as both concepts are distinct but similar. Pearlin et al. [19] discovered that social support, such as at the workplace, could reduce the impact of involuntary job disruptions on mental well-being. Workplaces with more trust, understanding, and common values can possibly better suit the needs of returning workers after cancer. Previous studies explored these needs as well as other factors, including vulnerability, understanding, and the need for support [35, 36]. Furthermore, a recent intervention designed to support employers after cancer established communication between employers and employees by helping them understand the survivors’ situation by informing and considering different cancer “experience types” [37]. These aspects might already be more pronounced in workplaces with higher levels of social capital, increasing BCSs’ satisfaction with the occupational development. The SOCAPO-E instrument was designed to measure social capital according to the evaluations of many employees and validated for healthcare organizations. In the present study, the instrument was appropriately used for single social capital evaluations per workplace, with Cronbach’s alpha of 0.94.

The comparison of the results between the imputed data (M1a–M3a) and the nonimputed data (M1b-M3b) revealed similar regression coefficients, except for variables such as marital status, number of children, vocational training, and recurrence. For these variables, the coefficients were higher in the nonimputed results, possibly indicating overestimation.

### Strengths and limitations

To our knowledge, this study is the first to focus on the occurrence of involuntary job changes and explore the associations between job changes, involuntariness, and satisfaction with the occupational development 5–6 years after breast cancer diagnosis, while controlling for sociodemographic, disease-related, and work(place)-related variables.

The longitudinal design considers numerous sociodemographic as well as disease-related and work(place)-related influencing factors. The measurement time points covered different stages of the cancer journey, starting from acute therapy until the 5-year survivor phase.

The B-CARE study applied an observational approach. Hence, only associations and not causal relations could be described. The study design hinders the comparison of job patterns between BCSs and healthy women. Therefore, we could not evaluate whether BCSs’ experiences differed in terms of the number of job changes and their involuntariness. Nevertheless, the results showed that involuntariness occurred and that support is needed to aid BCSs’ ability to work as desired. Future research may include a healthy comparison group.

Considering the design of the PIAT and B-CARE projects, which required respondents to answer surveys at several measurement time points, a bias in the sample toward BCSs with better physical and mental health status was possible. Assuming the occurrence of this bias, variables, such as subjective health status, might have been underestimated and actually lower, while the satisfaction with the occupational development might have been overestimated. Furthermore, in the original sample and the analyzed subsample, more motivated and educated people and those with sufficient language skills were likely overrepresented. Therefore, individuals with more precarious employment situations or those with a migration background were possibly underrepresented.

Recall bias could affect the retrospective variables measured at T4 that referred to RTW (e.g., the social capital of the workplace where the BCSs returned to). Moreover, the dependent variable of satisfaction with the occupational development included a single nonvalidated item wherein participants were asked to evaluate the time span of 5–6 years prior; therefore, this could be vulnerable to a recall bias or be influenced by recent events. However, during the pretests, no problems in recalling this information were noted. Satisfaction with the occupational development was measured at T4 only; therefore, we could not rule out the existence of differences in satisfaction with the occupational development before the diagnosis because we did not compare previous satisfaction with the occupational development before and after the diagnosis. All measured job changes were included in the analyses equally. However, the bi-directionality (increase/decrease) of some changes could potentially impact the satisfaction with the occupational development because some changes may be generally regarded as positive or negative. For instance, an increase in payment could be considered a positive change. However, the present results showed that BCSs’ involuntariness ratings of job changes are complex, similar to the increase in payment, which was rated as (rather) involuntary (Table 2). Additionally, a recent qualitative study indicates that job changes, even when financially disadvantageous, are welcomed by male BCSs [38].

In addition, the sample size in this study was rather small, indicating limited statistical power.

## Conclusion

Job changes alone were not substantially associated with BCSs’ satisfaction with the occupational development 5–6 years after diagnosis. However, experiencing involuntary job changes is associated with lower levels of BCSs’ satisfaction with their occupational development. Thus, long-term support aiming at strengthening the work ability is essential to prevent BCSs from experiencing involuntariness and enable their ability to work as desired. The significance of workplace characteristics highlights the need for employers to encourage satisfying work participation. Involuntariness is an important measure to understand how disadvantageous work-related outcomes are and to determine the need for support. For a more distinct understanding of BCSs’ long-term work-related outcomes, conducting more research on subjective work-related outcomes is necessary.

## Supplementary Information

ESM 1 (DOCX 14 kb).

## Data Availability

According to the patient consent form, data is not available for scientific use by others, other than the project group members.
