# Supplementary material for: Involuntariness of job changes is related to less satisfaction with occupational development in long-term breast cancer survivors
Source: J Cancer Surviv. 2021 Apr 27;16(2):397–407. doi: 10.1007/s11764-021-01035-5 (PMC8964540; doi:10.1007/s11764-021-01035-5)
Supplement: Supplementary file 1 — (DOCX 14 kb). [file 11764_2021_1035_MOESM1_ESM.docx]

**Supplementary material**

**Table S1:** Excerpt from the questionnaire: Measurement of job changes and involuntariness rating

|  | Changes when returning to work | Further changes (1) | Further changes (2) | Further changes (3) |
| --- | --- | --- | --- | --- |
| Which occupational changes (including all consequences) have occurred? Multiple choice is possible. | - Decreased working time - Increased working time - Decreased scope of work - Increased scope of work - Decreased work load - Increased work load - Decreased payment - Increased payment - Change of employer - Change within employer - Retirement:   - caused by age   - early retirement   - reduced earning capacity (partial)   - reduced earning capacity (complete) | - Decreased working time - Increased working time - Decreased scope of work - Increased scope of work - Decreased work load - Increased work load - Decreased payment - Increased payment - Change of employer - Change within employer - Retirement:   - caused by age   - early retirement   - reduced earning capacity (partial)   - reduced earning capacity (complete) | - Decreased working time - Increased working time - Decreased scope of work - Increased scope of work - Decreased work load - Increased work load - Decreased payment - Increased payment - Change of employer - Change within employer - Retirement:   - caused by age   - early retirement   - reduced earning capacity (partial)   - reduced earning capacity (complete) | - Decreased working time - Increased working time - Decreased scope of work - Increased scope of work - Decreased work load - Increased work load - Decreased payment - Increased payment - Change of employer - Change within employer - Retirement:   - caused by age   - early retirement   - reduced earning capacity (partial)   - reduced earning capacity (complete) |
| How satisfied were you with these occupational changes? Single choice. | - Unsatisfied - Rather unsatisfied - Partly - Rather satisfied - Satisfied | - Unsatisfied - Rather unsatisfied - Partly - Rather satisfied - Satisfied | - Unsatisfied - Rather unsatisfied - Partly - Rather satisfied - Satisfied | - Unsatisfied - Rather unsatisfied - Partly - Rather satisfied - Satisfied |
| How voluntary were these occupational changes? Single choice. | - Involuntary - Rather involuntary - Partly - Rather voluntary - Voluntary | - Involuntary - Rather involuntary - Partly - Rather voluntary - Voluntary | - Involuntary - Rather involuntary - Partly - Rather voluntary - Voluntary | - Involuntary - Rather involuntary - Partly - Rather voluntary - Voluntary |
| When did the occupational changes occur approximately? | ___ (MM) /______(YYYY) | ___ (MM) /______(YYYY) | ___ (MM) /______(YYYY) | ___ (MM) /______(YYYY) |
